# Central Texas Medical Association, 20th Quarterly Session

**Published:** 1891-06

**Authors:** W. D. Wilkes

**Affiliations:** Secretary; Waco, Texas


### Central Texas Medical Association.

WACO, Texas, May 9th, 1892.

DEAR DOCTOR—You are cordially invited to attend the twentieth quarterly session of the Central Texas Medical Association, in Waco, Tuesday, July 14th, 1891.

PROGRAMME.

1. "La Grippe and Its Sequelæ," by Dr. G. B. Foscue, of Waco; discussion to be opened by Drs. W. A. Howard, of Waco, and H. M. Steward, of Patrick.
2. "Rheumatism," by Dr. R. C. Nettles, of Marlin; discussion to be opened by Drs. R. W. Park, of Waco, and J. C. Shaw, of Stranger.
3. "Treatment of Compound fractures of the Cranium," by Dr. J. C. J. King, of Waco; discussion to be opened by Drs. W. M. Drake, of Hillsboro, and W. R. Blailock, of McGregor.
4. "The Treatment of Tubercle of the Bones," by Dr. W.

E. Brown, of Gatesville; discussion to be opened by Drs. J. W. Miller, of Itasca, and J. M. Woodson, of Temple.

5. "What is a Tonic?" by Dr. J. H. Sears, of Waco; discussion to be opened by Drs. M. L. Graves, of Georgetown, and W. M. Shankee, of Chilton.

W. D. WILKES, M. O., Secretary.

---

**What Should be Done in Gunshot Wounds of the Abdomen.**—Lamphear, of Kansas City, in the *Medical Review* of June 6, 1891, makes answer to this question in one word—operate! After a most careful study of the statistics, he finds that under the old method of treatment (the "expectant plan") the mortality rate was 88 per cent.; but under the "American method" of treatment (laparotomy and irrigation) the death rate has been reduced to nearly 60 per cent. Death, he claims, may be due to (1) hemorrhage, (2) perforation of the intestine or other viscera, (3) peritonitis, (4) septicæmia—in any one of which death may be prevented by operation. In gunshot wounds of the abdomen, Senn's hydrogen gas test should not be used, as the indications are always to operate, because perforation of intestine is not at all necessary to cause death—in fact, the majority of cases will die of hemorrhage or of septicæmia if no laparotomy be made. Rents in the bowels are to be sewed up, and buried by the Lambert suture, or an astomosis made if there be great destruction of tissue. As much as six feet of intestine may be removed without death. For making the Lambert suture, only the finest "milliners' silk" is to be used, with a fine cambric needle. Irrigation must be employed if there have been fœcal extravasation, if septic material have been introduced from any source, or if there be much peritonitis. If not, the blood is simply sponged out carefully (after all bleeding points have been secured), and the abdominal incision closed as in any laparotomy. While the best results are obtained within twelve hours after the reception of the injury, the passage of several days need not prevent a successful issue if operation be made. Peritonitis demands, rather than contraindicates, operation.

